# A benchmarking method to evaluate the accuracy of a commercial proton monte carlo pencil beam scanning treatment planning system

**DOI:** 10.1002/acm2.12043

**Published:** 2017-02-02

**Authors:** Liyong Lin, Sheng Huang, Minglei Kang, Petri Hiltunen, Reynald Vanderstraeten, Jari Lindberg, Sami Siljamaki, Todd Wareing, Ian Davis, Allen Barnett, John McGhee, Charles B. Simone, Timothy D. Solberg, James E. McDonough, Christopher Ainsley

**Affiliations:** ^1^ Department of Radiation Oncology University of Pennsylvania Philadelphia Pennsylvania USA; ^2^ Varian Medical Systems Palo Alto California USA

**Keywords:** AcurosPT, commissioning, Monte Carlo dose calculation, pencil beam scanning, proton therapy

## Abstract

AcurosPT is a Monte Carlo algorithm in the Eclipse 13.7 treatment planning system, which is designed to provide rapid and accurate dose calculations for proton therapy. Computational run‐time in minimized by simplifying or eliminating less significant physics processes. In this article, the accuracy of AcurosPT was benchmarked against both measurement and an independent MC calculation, TOPAS. Such a method can be applied to any new MC calculation for the detection of potential inaccuracies. To validate multiple Coulomb scattering (MCS) which affects primary beam broadening, single spot profiles in a Solidwater^®^ phantom were compared for beams of five selected proton energies between AcurosPT, measurement and TOPAS. The spot Gaussian sigma in AcurosPT was found to increase faster with depth than both measurement and TOPAS, suggesting that the MCS algorithm in AcurosPT overestimates the scattering effect. To validate AcurosPT modeling of the halo component beyond primary beam broadening, field size factors (FSF) were compared for multi‐spot profiles measured in a water phantom. The FSF for small field sizes were found to disagree with measurement, with the disagreement increasing with depth. Conversely, TOPAS simulations of the same FSF consistently agreed with measurement to within 1.5%. The disagreement in absolute dose between AcurosPT and measurement was smaller than 2% at the mid‐range depth of multi‐energy beams. While AcurosPT calculates acceptable dose distributions for typical clinical beams, users are cautioned of potentially larger errors at distal depths due to overestimated MCS and halo implementation.

## 1 Introduction

For pencil beam scanning (PBS), Monte‐Carlo (MC) based treatment planning systems (TPSs)^1^, ^2^, ^3^ can potentially calculate dose distributions more accurately than those based on analytical algorithms.^4^ Full MC calculations, such as Geant4^5^ and MCNPX2.7,^6^ often take hours to achieve satisfactory statistical uncertainty for PBS plans, which is unacceptable for clinical application. A fast MC calculation module, AcurosPT with simplified radiation transport, has been benchmarked with MCNPX 2.7 and released in a commercial TPS, Eclipse 13.7 (Varian Medical Systems, Palo Alto, CA). Like AcurosXB,^7^ AcurosPT uses a form of Fokker‐Planck approximation for multiple Coulomb scattering (MCS).^8^ In contrast with AcurosXB, however, which is a deterministic algorithm for coupled photon and electron transport problems, AcurosPT calculates dose distributions by direct MC simulation of PBS proton transport. A parallel implementation as well as several approximations enables AcurosPT to calculate the dose for a typical PBS treatment plan within a few minutes, yielding 2% statistical uncertainty of the target dose.

To achieve the required MC dose calculation accuracy, both the proton source and radiation transport mechanisms must be appropriately implemented so that proton spot profiles can be correctly calculated from primary and halo regions for multi‐spot PBS plans.^9^, ^10^ On one hand, primary spot profiles are affected by the choice of MCS mechanism.^11^, ^12^ Conversely, the halo is predominantly caused by large angle scattering and nuclear interactions within the phantom, in addition to that from the nozzle (i.e. proton source). The halo can affect the output of proton beams by up to 10% depending on field size, proton energy and depth.^9^, ^10^, ^13^ Therefore, in commissioning a new MC calculation module, it is essential to evaluate clinically relevant scenarios for both single and multiple spot profiles.

AcurosPT utilizes a simplified radiation transport to improve calculation efficiency. AcurosPT was internally benchmarked for agreement with MCNPX 2.7, as there was no measurement data available to the TPS vendor during the development of new MC calculation module. In that regard, Stankovskiy et al.^14^ have reported underestimation of Bragg peak curves using fast Monte Carlo and benchmarking with MCNPX.^15^ reported that calculated spot profiles are larger in MCNPX than in Geant4, while Sawakuchi et al.^16^ have reported the overestimation of spot profiles in MCNPX compared with measurement.

The modeling of radiation transport affects the dosimetric agreement for most proton energies. For low proton energies (below 150 MeV), Lin et al.^17^ pointed out that for some treatment nozzles, the halo from the nozzle can be more dominant than that in phantom. The treatment nozzle halo can be modeled in Monte Carlo based on nozzle design^16^ or using experimental data at nozzle exit.^18^ These user defined parameters are vital to the dosimetric agreement for low proton energies. Prior publications have stressed the importance of modeling radiation transport in phantom and proton source. In this paper, we assess the accuracy of the AcurosPT dose calculation algorithm and the validity of the proton source by benchmarking AcurosPT against both measurement and an independent Monte Carlo dose calculation^19^, ^20^ TOPAS,^21^ that is based on Geant4. This benchmark process is broadly applicable and can be utilized to identify potential inaccuracies in any newly released MC‐based TPS during commissioning.

## 2 Methods and materials

The IBA dedicated PBS nozzle and its design used in this study have been previously described.^17^, ^22^ The nozzle was characterized in the Eclipse TPS with the AcurosPT 13.7 calculation algorithm, which uses interaction data from MCNPX2.7. AcurosPT 13.7 is a Monte Carlo algorithm designed to provide rapid and accurate dose calculations for proton radiotherapy problems by simplifying or eliminating less significant physics processes to keep the computational run‐time to a minimum. AcurosPT simplifies radiation transport into four categories: slowing down interactions with atomic electrons, elastic nuclear Coulomb scattering, elastic nuclear strong‐force scattering, and non‐elastic nuclear reactions. Decisions as to what simplifications are appropriate and what physics to include are validated by comparison of the results with more detailed calculations with Acuros PT itself and with MCNPX^23^ and T. Wareing, P. Hiltunen and R. Vanderstraeten, personal communication. The Double Gaussian fluence model^24^, ^25^ was implemented in AcurosPT using a phase space approach.^26^ The phase space approach uses reference beam measurement data to derive the beam optics parameters and therefore provides a more accurate derivation of spot sigma than a non‐phase space approach for non‐reference proton energies and in‐air locations. The use of independent Monte Carlo simulation platforms can help determine the cause of any dose differences with respect to measurements; i.e. whether the differences are due to simplified radiation transport approximations made to speed up the calculations in the phantom, or the user‐defined PBS source model. Thus, to understand the residual disagreement between AcurosPT and measurement, independent TOPAS simulations were set up for the same scenarios.^18^ The double Gaussian phase space source model parameters were optimized in AcurosPT 13.7 and TOPAS 2.0 to match the measurement data. For the TOPAS simulations, the default Physics list, which includes G4EMStandardPhysics_option3, HadronPhysicsQGSP_BIC_HP, G4IonBinaryCascadePhysics and G4HadronElasticPhysicsHP models, was used. The derivation of proton source parameters have been reported previously.^18^

To commission AcurosPT, fifteen integral Bragg peaks, collected using a PTW‐34070 Bragg peak chamber (Freiburg, Germany), for a total of 15 corresponding proton energies from 100 MeV to 220 MeV in 10 MeV steps plus 115 and 225 MeV, were provided. AcurosPT calculates the number of protons per monitor unit (MU), the energy spread in MeV and the mean energy for each of these 15 nominal energies. These AcurosPT provided data were independently checked and adjusted to better fit measurement.

A Lynx^®^ device,^27^ a scintillation screen/CCD camera detector made by IBA Dosimetry (Schwarzenbruck, Germany), which has a spatial resolution of 0.5 mm, was used to measure in‐air profiles of single and multi‐spot fields. Single‐spot profiles for five selected nominal energies were validated, as well as multi‐spot profiles of multi‐energy PBS beams (RxMyFz with range R of x mm, modulation M of y mm and square field size F of z mm), at different depths in Solidwater^®^ (Gammex, Inc., Wisconsin, USA). In this article, ‘spot sigma’, which refers to one standard deviation of the Gaussian spatial spread, is used to describe the spot profile. The uncertainty on the spot sigma values were derived from measurements repeated on three different days. In multi‐spot beams, 1 MU^1^ per spot was used and spots were uniformly spaced 4 mm apart at isocenter. TOPAS simulations of multi‐spot beams used 0.1–0.3 million protons per spot using the same 1 mm calculation grids as used for AcurosPT. In multi‐energy beams, a minimal numbers of energies were used to achieve ± 1% dose uniformity within the modulation length. Due to concern over the scintillator's energy‐dependent response,^28^ Lynx measurements were limited to relative dose distributions that involve minimal energy variation.

Absolute output and depth doses were validated in a water phantom, depth by depth, using a MatriXX PT^®^, which utilizes a new two‐dimensional ionization chamber array designed specifically for the high dose rates in PBS delivery.^29^ Agreement of field size factor (FSF) calculations with measurements, where FSF is defined to be the output at the center of a given square field size^2^ relative to that for a field size of 96 mm, was used to quantify the accuracy of the halo characterization^25^ in AcurosPT for different nominal proton energies. Absolute depth dose and profile measurements were validated for multi‐energy beams RxMyFz, to reduce the measurement uncertainty along the depth direction. FSF analysis at selected proton energies aids in the interpretation of radiation transport inaccuracy related to proton energy.

## 3 Results

**Figure 1:**
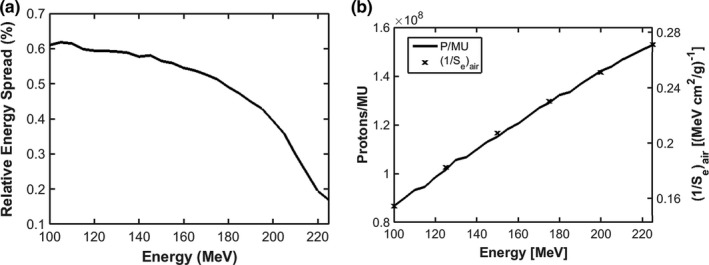
Gaussian energy spread (a) and number of protons per MU and the inverse of electronic proton stopping power in air (b) vs. proton energy for the IBA dedicated PBS nozzle.

Fig. 1 shows the variation in Gaussian energy spread and number of protons per MU with proton energy. For our system, the energy spread decreases from above 0.6% at 100 MeV to below 0.2% at 225 MeV, similar to that reported by.^15^ The number of protons per MU increases with proton energy from ~8.5E7 at 100 MeV to ~1.55E8 at 225 MeV. The number of protons per MU is approximately proportional to the electronic proton stopping power within 1%.^30^, ^31^

**Figure 2:**
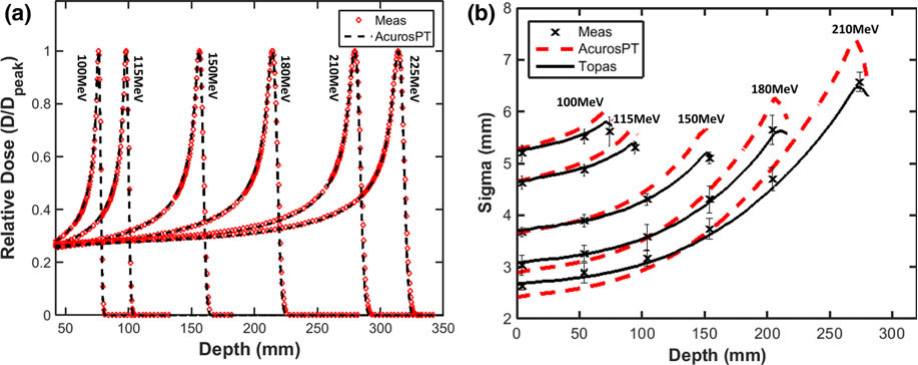
Comparison of simulated Bragg peaks between AcurosPT and measurement (a) and spot sigma of single pencil beams as a function of depth in Solidwater^®^ phantom (b).

Fig. 2(a) shows the comparison of Bragg peaks between AcruosPT and measurement. The relative dose agreement between AcurosPT and measurement is greater than 99% using gamma criteria of 1 mm/1% for the pass rate, for all the Bragg peaks except some marginal 4% failures for the lowest 100 MeV beam (Fig. 2(a)), validating the derived energy spread in Fig. 1(a). The TOPAS simulation is indistinguishable from measurement and therefore omitted from the figure. In contrast, Fig. 2(b) indicates that AcurosPT calculates too large a spot sigma, which increases with depth in comparison to measurement, leading to the largest disagreement near the end of the proton range. The disagreement can be up to 15% (maximum 1 mm) larger than measurement, which is larger than the measurement uncertainty. Allowing AcurosPT's spot sigma to be smaller than measurement at phantom entrance, this disagreement at all depths is comparable, however, to the tolerance of 10% or 1 mm recommended by^32^ and 15% observed by,^33^ and smaller than the 20% day‐to‐day variation specified by our proton therapy treatment delivery vendor. In contrast, the spot sigma calculated by TOPAS followed measurement much better than AcurosPT and was well within the measurement uncertainty.

**Figure 3:**
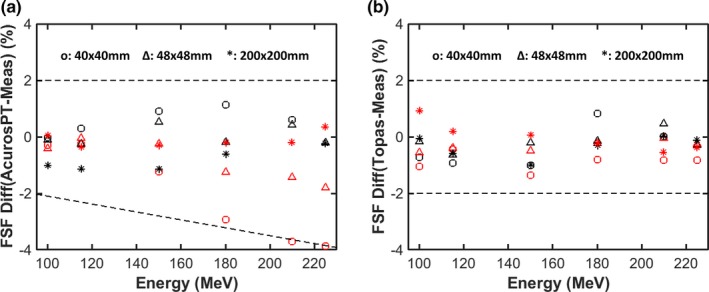
Percentage differences between the calculated and measured field size factors (FSF) for three field sizes at two depths as a function of proton energy for AcurosPT (a) and TOPAS (b). The black markers represent the results at surface while the red markers represent depths close to the Bragg peak. The dashed lines are used for visual guidance of large FSF disagreements.

Fig. 3(a) shows that AcurosPT also tends to underestimate FSFs. The underestimation of FSF can reach 4% at the deeper depth for a 40 mm field size when the proton energy exceeds 200 MeV. This is due to the overestimation of MCS and halo magnitude, which increases with depth in AcurosPT. This overestimation becomes reduced at a field size of 48 mm and is eventually undetectable at a field size of 200 mm. Because TOPAS‐simulated FSFs agreed with measurement mostly within 1% for the same conditions (Fig. 3(b)), it is unlikely this phenomenon is due to measurement error. Despite the above overestimation of MCS and halo magnitude in phantom, both AcurosPT and TOPAS agreed with measurement within 1.5%. This is because FSFs are predominantly determined by user defined source parameters for proton energies below 150 MeV.^17^

## 4 Discussion

To better distinguish the impact on penumbra and absolute dose for more typical clinical conditions, multiple proton energies were used (from 103.3 to 128.3 MeV for R120M40, from 116.1 to 172.3 MeV for R200M100 and from 178.5 to 220.5 MeV for R305M100) to achieve a uniform dose along the prescribed modulation length with a field size of 96 mm.

**Figure 4:**
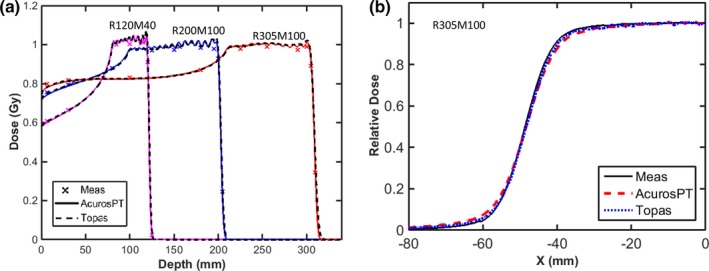
(a): The central axis depth doses calculated by AcurosPT (solid line) and TOPAS (dashed line) are compared with measurements (cross‐marker) for three proton beams of different proton ranges and modulation width of 100 or 40 mm with field size of 96 mm. Depth dose curve of R120M40 was renormalized by multiplying 105% to avoid overlap with R200M100. (b): The lateral dose profile at mid‐range depth of the R305M100 beam.

Fig. 4(a) shows the agreement of absolute dose between AcurosPT and measurement, within 1 mm/2% for all of the beams studied. This good agreement is expected, as the output variation caused by halo modeling becomes smaller with larger field sizes. The very good agreement within 0.5 mm/0.5% between TOPAS and AcurosPT indicates that the residual disagreement between AcurosPT and measurement over the buildup or ripple/uniform regions are more likely due to measurement uncertainties rather than calculation inaccuracy in AcurosPT.

Since the overestimation of spot sigma in AcurosPT is larger for more distal depths of high energy proton beams, the penumbra of a multi‐energy beam has a larger difference at a mid‐SOBP depth of 255 mm for R305M100 than at mid‐SOBP depths of 150 mm for R200M100 and 100 mm for R120M40 (Table 1). The agreement of penumbra at these depths is within 1 mm/1% with the exception of the 255 mm depth that can have a detectable difference between 1 mm/1% and 2 mm/2%. Table 1 and Fig. 4(b) also show that the distance‐to‐agreement of profiles at 95% and 5% of the uniform dose can exceed 2 mm with dose difference within 2%.

**Table 1:** Comparison of dosimetric parameters of lateral dose profiles at mid‐range of SOBPs in a Solidwater^®^ phantom

SOBP	Penumbra 20%–80% (mm)			Half‐width of 95% shoulder (mm)			Half‐width of 5% shoulder (mm)		
	Meas	AcurosPT	Topas	Meas	AcurosPT	Topas	Meas	AcurosPT	Topas
R120M40	8.0	8.3	8.3	41.7	40.8	40.8	58.3	59.2	59.2
R200M100	7.8	8.1	8.0	39.7	38.5	39.2	56.5	57.9	57.3
R305M100	10.2	11.8	10.7	37.4	34.6	36.0	59.8	63.3	61.0

## 5 Conclusion

In this article, we describe a benchmark method to detect potential radiation transport/proton source inaccuracies in a commercial MC TPS using measurement and an independent MC calculation. Using such a method, we detect the MCS and halo overestimation in AcurosPT, which can be traced back to MCNPX. Benchmarking the TPS to standard MC platforms alone might not be sufficient for a commercial release of fast MC calculation. AcurosPT can calculate acceptable dose distribution for typical clinical proton beams within 2 mm/2%, though caution may be warranted at very distal depths where small field's FSF might be underestimated by approximately 4%.

## Acknowledgment

Liyong Lin thank Chris Beltran from Mayo Clinic for his suggestions regarding AcurosPT.

## Conflicts of interests

Liyong Lin has a 2‐yr grant under the master agreement between University of Pennsylvania and Varian Medical Systems. There are no other relevant conflicts of interest to disclose.

## Endnotes

1 In the IBA definition, 1 MU corresponds to 3 nC collected in a 1 cm gap air‐filled ion chamber.

2 Average output at central four detectors, which are 7.6 mm aside from each other.
